# Apathy Is Correlated with Widespread Diffusion Tensor Imaging (DTI) Impairment in Amyotrophic Lateral Sclerosis

**DOI:** 10.1155/2018/2635202

**Published:** 2018-10-22

**Authors:** Cinzia Femiano, Francesca Trojsi, Giuseppina Caiazzo, Mattia Siciliano, Carla Passaniti, Antonio Russo, Alvino Bisecco, Mario Cirillo, Maria Rosaria Monsurrò, Fabrizio Esposito, Gioacchino Tedeschi, Gabriella Santangelo

**Affiliations:** ^1^Department of Medical, Surgical, Neurological, Metabolic and Aging Sciences; MRI Research Center SUN-FISM, Università degli Studi della Campania “Luigi Vanvitelli”, Naples, Italy; ^2^Department of Psychology, Università degli Studi della Campania “Luigi Vanvitelli”, Naples, Italy; ^3^Department of Medicine, Surgery and Dentistry, Scuola Medica Salernitana, University of Salerno, Baronissi, Salerno, Italy

## Abstract

Apathy is recognized as the most common behavioral change in several neurodegenerative diseases, including amyotrophic lateral sclerosis (ALS), a multisystem neurodegenerative disorder. Particularly, apathy has been reported to be associated with poor ALS prognosis. However, the brain microstructural correlates of this behavioral symptom, reported as the most common in ALS, have not been completely elucidated. Using diffusion tensor imaging (DTI) and tract-based spatial statistics (TBSS), here we aimed to quantify the correlation between brain microstructural damage and apathy scores in the early stages of ALS. Twenty-one consecutive ALS patients, in King's clinical stage 1 or 2, and 19 age- and sex-matched healthy controls (HCs) underwent magnetic resonance imaging and neuropsychological examination. Between-group comparisons did not show any significant difference on cognitive and behavioral variables. When compared to HCs, ALS patients exhibited a decreased fractional anisotropy (FA) [*p* < .05, threshold-free cluster enhancement (TFCE) corrected] in the corpus callosum and in bilateral anterior cingulate cortices. Self-rated Apathy Evaluation Scale (AES) scores and self-rated apathy *T*-scores of the Frontal Systems Behavior (FrSBe) scale were found inversely correlated to FA measures (*p* < .05, TFCE corrected) in widespread white matter (WM) areas, including several associative fiber tracts in the frontal, temporal, and parietal lobes. These results point towards an early microstructural degeneration of brain areas biologically involved in cognition and behavior regulation in ALS. Moreover, the significant correlations between apathy and DTI measures in several brain areas may suggest that subtle WM changes may be associated with mild behavioral symptoms in ALS even in the absence of overt cognitive and behavioral impairment.

## 1. Introduction

Apathy is defined as decreased motivation towards goal-directed behaviors [1] and may occur as a symptom of a variety of different psychiatric and neurodegenerative diseases [2, 3]. In particular, several studies have revealed that apathy is the most common behavioral symptom in amyotrophic lateral sclerosis (ALS) [4–6], a multisystem neurodegenerative disease that mainly affects motor areas and exhibits cognitive and behavioral symptoms belonging to the spectrum of frontotemporal lobar degeneration (FTLD) syndrome [7, 8]. Of note, while approximately 5–15% of ALS patients meet the diagnostic criteria for FTLD [9, 10], predominantly developing clinical features typical of the behavioral variant frontotemporal dementia (bvFTD) [10], most patients exhibit behavior and/or cognitive dysfunctions (i.e., ALS with behavioral impairment [ALSbi], with cognitive impairment [ALSci], and with both [ALSbci]), which are not sufficient to diagnose FTLD [8].

From the prognostic point of view, several studies demonstrated that ALS-FTLD patients have a significantly shorter survival than nondemented ones [11–13] and that behavioral impairment rather than dementia or cognitive impairment alone is an important predictor of survival in ALS patients [14]. Among behavioral disturbances, apathy may precede motor symptoms [15], represents an independent (negative) prognostic factor in ALS [16], and may even impact the ability to engage competently in end-of-life decisions [14, 17].

The most common instruments used to detect apathy in ALS patients are the Frontal Systems Behavior (FrSBe) scale, which includes an apathy subscale [18, 19]; the Dimensional Apathy Scale (DAS) [20]; and the Apathy Evaluation Scale (AES) [21]. While FrSBe is not specifically designed for testing patients with physical disability [18, 19], AES is a well-established method for assessing apathy in several neurodegenerative diseases [3, 22]. Both FrSBe and AES allow performing a monodimensional evaluation of apathy. However, this symptom is actually composed of multiple factors related to the cognitive, behavioral, and emotional domains [23], and recent studies employed a factorial analysis of AES scores revealing a triadic or four-factor substructure of the scale in Parkinson's disease [24, 25].

From the neuroanatomical point of view, some structural correlates of behavioral changes have been identified in frontosubcortical circuits in healthy subjects as well as in ALS patients [26, 27] using neuropsychological and advanced magnetic resonance imaging (MRI) evaluations. In this regard, diffusion tensor imaging (DTI) is the advanced MRI method of choice to investigate *in vivo* the integrity of large white matter (WM) tracts in terms of fractional anisotropy (FA) and mean (MD), axial (AD), and radial (RD) diffusivity parameters [28, 29]. Particularly, DTI allowed exploring the structural connectivity in several neurodegenerative diseases, including Alzheimer's disease [30] and the disease spectrum of ALS-FTLD [31–37].

So far, only a few studies have explored the brain structural correlates of apathy in ALS, suggesting that WM abnormalities within anterior cingulum bundle are the most relevant substrates of apathy in ALS [36, 37]. However, in those studies, apathy was only evaluated by the subscale of FrSBe, and patients in early disease stages, according to validated staging systems, were not included.

On this background, using a tract-based spatial statistics (TBSS) DTI approach [38], we aimed to explore the potential association between brain microstructural damage and apathy, using both self- and informant-rated version of the AES, and by the apathy subscale of FrSBe, in the early stages of ALS according to a validated staging system for ALS.

## 2. Methods

### 2.1. Case Selection

Twenty-two right-handed patients (13 men, 9 women; mean age 58.19 ± 9.63), 14 with definite ALS and 8 with probable or probable laboratory-supported ALS, according to the revised El Escorial criteria [39], were consecutively recruited at the First Division of Neurology of the University of Campania “Luigi Vanvitelli” (Naples, Italy). Genetic analysis was performed in all patients, exploring *C9ORF72* expansion and mutations of *superoxide dismutase 1* (*SOD1*), *TAR DNA binding protein* (*TARDBP*), and *fused in sarcoma/translocated in liposarcoma* (*FUS*/*TLS*). Only a woman with familial ALS, carrier of *FUS/TLS* gene mutation (codon 1622 G>A), was not included in the analysis. With regard to clinical phenotypes, according to the ALS subtype classification of Chiò et al. [40], 9 patients had a bulbar onset and 13 a spinal onset; disease phenotypes were classic (*n* = 8), bulbar (*n* = 4), flail arm (*n* = 2), flail leg (*n* = 1), and pyramidal (*n* = 7). Patients were classified according to King's clinical staging system for ALS [41] as follows: Stage 1: involvement of a first region; Stage 2: involvement of a second region; Stage 3: involvement of a third region; and Stage 4: need for gastrostomy or respiratory support (noninvasive ventilation). All the patients enrolled were in King's clinical stage 1 or 2.

As for clinical parameters, we administered the ALS functional rating scale-revised (ALSFRS-R) score, an index of disability status [42], and the Upper Motor Neuron (UMN) score, a measure of pyramidal dysfunction through the evaluation of the number of pathologic reflexes elicited from 16 body sites (i.e., one-sided, glabellum and masseter, and, bilaterally, orbicularis oris, biceps, triceps, finger jerk, knee, ankle, and Babinski responses) [43]. Moreover, we also estimated the disease progression rate according to the following formula: 48 − ALSFRS-R score/months of disease duration [44]. None of the patients had additional neurological diseases or previous mental illnesses.

Nineteen right-handed healthy control subjects (HCs) (9 men, 10 women; mean age 57.53 ± 9.03) were enrolled by “word of mouth” among (nonphysician) employees at the University (*n* = 10) or friends and colleagues of patients' caregivers (*n* = 9). HCs were cognitively normal and had no comorbid neurological, psychiatric, or medical conditions; moreover, they were age-, sex- and education-matched with the enrolled ALS patients (Table 1).

### 2.2. Neuropsychological Assessment

A 60 min neuropsychological battery, assessing cognitive functioning/ability (global cognitive assessment and executive, memory, and visuo-spatial abilities) and frontal behavioral disorders, was designed by a team of neurologists and neuropsychologists experienced in the study and management of motor neuron diseases and cognitive decline. Although this neuropsychological battery was not ALS-specific, the tests administered have been widely used in ALS and other movement disorders [45–48]. All tests were administered in the morning following the same sequence to avoid possible interference of the answers of one test over the others [47]. Moreover, if the subject was tired during testing, a further session was scheduled to complete the battery within two weeks from the first one. Considering that respiratory dysfunction may affect cognitive performances [49–51], oxygen saturation and forced vital capacity (FVC) were measured at the time of each examination (i.e., no participants showed oxygen saturation < 92 mmHg and FVC < 80%) [47].

#### 2.2.1. Cognition

The following cognitive domains were evaluated in both ALS patients and HCs: (1) global cognitive impairment by Mini-Mental State Examination (MMSE); (2) executive functions (i.e., the inhibitory control) by the Stroop Color-Word Interference test using the Stroop Executive Factor (SEF) [52], which accounts for motor disability [48], and by the forward and backward digit span tasks [53], assessing working memory and sustained attention; (3) language (i.e., verbal comprehension abilities), by Token Test [54]; (4) long-term verbal memory, by memory prose test [55]; (5) nonverbal abstract reasoning by Raven's Colored Progressive Matrices (RCPM) [56]; and (6) visuo-spatial abilities by visual discrimination (scrawls) test [54].

#### 2.2.2. Neurobehavioral Aspects

Apathy was evaluated by both self-rated and informant-rated versions of the apathy subscale of the FrSBe [18, 19] and AES scales (S-AES and I-AES) [21]. As for FrSBe (score range: 46–230 points for total score; 14–70 points for apathy subscale), the raw scores were converted to *T*-scores according to gender, age, and years of education [19]. *T*-Scores were considered as “borderline” impaired when ranging from 60 to 64, while *T*-scores higher than or equal to 65 reflected pathological changes [57].

As for AES (score range: 18–72 points), we used a cut-off score of 41 for I-AES and 40 for S-AES. These clinical thresholds were estimated using descriptive statistics as reported in Marin et al. [21] and using 2 standard deviations above the mean as critical value. In addition to total score, the AES subscale scores (i.e., cognitive, behavioral, emotional, and other subscales) [21, 25] were employed for the analyses. All subjects completed the Beck Depression Inventory-II (BDI-II) [58], with score range from 0 to 63 points; the standard cut-off ranges were as follows: 0–9 indicated minimal depression, 10–18 indicated mild depression, 19–29 indicated moderate depression, and 30–63 indicated severe depression [58]. Overall, higher scores indicated more severe behavioral symptoms for all these scales.

### 2.3. Statistical Analysis: Between-Group Comparisons of Demographic, Clinical and Neuropsychological Data

Demographic, clinical, and neuropsychological data were reported for ALS patients and HCs. The Shapiro-Wilk test was used to assess normality and statistical dependency from the distribution of the data; *t*-tests, Mann-Whitney *U* tests, and chi-square tests (all Bonferroni corrected) were used to compare demographic, clinical, neuropsychological, and neurobehavioral scores between ALS patients and HCs. SPSS statistics 21.0 was used to perform all statistical analyses.

For more details about the correlations between apathy scores (i.e., S- and I-AES and apathy subscales of FrSBe) and whole-brain DTI measures, see paragraph on Section 2.6.

### 2.4. Ethics Statement

The research was conducted according to the principles expressed in the Declaration of Helsinki. Ethical approval was obtained from the Ethics Committee of the University of Campania “Luigi Vanvitelli” of Naples. Patient or family written consent was obtained from each participant.

### 2.5. Imaging Acquisition

Magnetic-resonance images were acquired on a 3 T scanner equipped with an 8-channel parallel head coil (General Electric Healthcare, Milwaukee, Wisconsin). Whole-brain DTI was performed using a GRE EPI sequence (repetition time = 10,000 ms, echo time = 88 ms, field of view = 320 mm, isotropic resolution = 2.5 mm, *b* value = 1000 s/mm^2^, 32 isotropically distributed gradients, and frequency-encoding RL).

### 2.6. Diffusion Tensor Imaging (DTI) Analysis

A voxel-based TBSS approach was used for the group analysis of DTI data [38]. DTI data sets were processed with the Functional MRI of the Brain (FMRIB) Software Library (FSL) software package (http://www.fmrib.ox.ac.uk/fsl), according to methods used in our previous studies [59, 60]. Preprocessing included eddy current and motion correction and brain-tissue extraction. After preprocessing, DTI images were averaged and concatenated into 33 (1 B = 0 + 32 B = 1000) volumes, and a diffusion tensor model was fitted at each voxel, generating AD, FA, MD, and eigenvalue (*λ*1, *λ*2, and *λ*3) maps. The average of the second and third eigenvalues of the diffusion tensor was used for the definition of RD. Images were warped to the Montreal Neurological Institute (MNI) 152 template, available as a standard T1 data set in the FSL software package. TBSS was run with FA maps to create the “skeleton,” which represents the center of all fiber bundles common to all subjects, and which was used for all other maps. To this purpose, FA images of all subjects (*n* = 40) were aligned to a common target (1 × 1 × 1 mm MNI152 FMRIB58_FA standard space) using nonlinear registration. A mean FA skeleton was then created with threshold of FA > 0.2. Age, gender, and education were considered as covariates. Moreover, the TBSS results were linked to standard anatomic data derived from the International Consortium of Brain Mapping DTI-81 WM label atlas (Johns Hopkins University, Baltimore, MD) [61, 62]. Individual skeleton images were submitted to a general linear model (GLM) analysis with appropriate design matrices and linear contrasts defined for the group comparisons and the correlations between all diffusivity parameters (FA, RD, MD, and AD) and clinical measures of apathy (score on self- and informant-rated AES, including the four factors of AES and *T*-scores of self- and informant-rated versions of the apathy subscale of the FrSBe) and depression (BDI-II scores). The results of voxel-wise correlations were shown on the skeleton map after correction for multiple comparison with the threshold-free cluster enhancement (TFCE) technique [38]. Age, gender, and education were considered as covariates, except for *T*-scores of the apathy subscale of the FrSBe.

## 3. Results

Demographic (age, gender, and education), clinical (disease duration, UMN scores, ALSFRS-R total score and subscores, and disease progression rate), neuropsychological, and neurobehavioral data of the ALS cohort (*n* = 21) and the HC group (*n* = 19) are reported in Table 1. Patients and HCs did not differ on demographic, cognitive, and behavioral parameters (Table 1). 4.7% of ALS patients (*n* = 1) and 5.2% of healthy subjects (*n* = 1) were above cut-off for apathy on the S-AES. Moreover, the same results were found when apathy was identified by the I-AES.

According to cut-off of BDI-II, 9 ALS patients and 11 healthy controls exhibited minimum depression; 9 ALS patients and 7 healthy controls, mild depression; and 3 ALS patients and 1 healthy control, moderate/severe depression.

### 3.1. TBSS DTI Analysis

**Figure 1:**
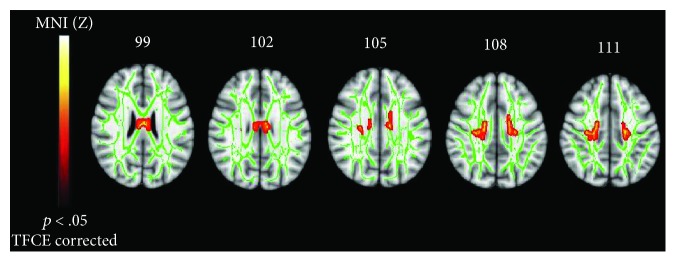
Comparison between FA statistic parametric maps of ALS patients versus HCs. FA decrease (red-yellow scale, *p* < .05, TCFE corrected; green: skeleton) is evident bilaterally in the midbody of the corpus callosum and in the anterior cingulum bundles.

Compared to HCs, ALS patients exhibited a decreased FA (*p* < .05, TFCE corrected) in the WM underneath the precentral gyri, in the body of the corpus callosum (CC) and in the bilateral anterior cingulate bundles (Figure 1), while no significant differences were observed in whole-brain MD, AD, and RD values.

### 3.2. Correlation between Apathy Scores and MRI Abnormalities

**Figure 2:**
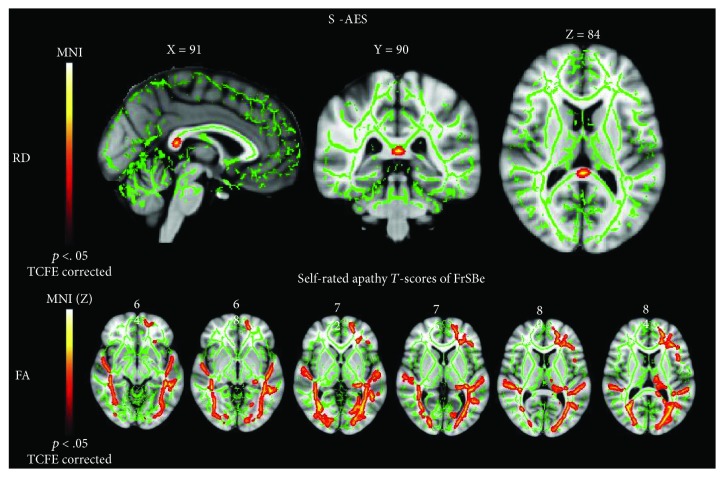
Voxel-wise correlation analyses between apathy scores (S-AES scores and self-rated apathy *T*-scores of FrSBe) and RD/FA in the studied ALS sample. Patterns of positive correlation (*p* < .05, TCFE corrected; red-yellow scale; green: skeleton) between S-AES scores and RD in the splenium of the CC and of negative correlation (*p* < .05, TCFE corrected; red-yellow scale; green: skeleton) between apathy *T*-scores of FrSBe and FA in several associative WM tracts in the patients' group.

S-AES scores were positively correlated with RD measures (Figure 2) (*p* < .05, TFCE corrected) in the splenium of the CC. Significant inverse correlations were found between the score on the self-rated version of the apathy subscore of FrSBe and FA measures (*p* < .05, TFCE corrected) in the left superior longitudinal fasciculus, in the right and left inferior longitudinal fasciculus, in the left fronto-occipital fasciculus, in the left uncinate fasciculus, in the left anterior cingulum bundle, and in the left thalamus (Figure 2). Moreover, positive correlations were revealed between the score on the self-rated version of the apathy subscore of FrSBe and RD measures (*p* < .05, TFCE corrected) in the splenium of the CC. Score on the informant-rated version of the apathy subscore of FrSBe was found to be inversely correlated with FA (*p* < .05, TFCE corrected) in the WM underneath the left temporal fusiform cortex. Finally, no significant correlation was reported between the BDI-II scores and the DTI measures and between the four factors (i.e., cognitive, behavioral, emotional, and other) of self-rated and informant-rated versions of the AES and the DTI metrics.

## 4. Discussion

Our findings revealed that ALS patients, in early stages of disease and without cognitive or behavioral impairment, may exhibit microstructural changes in both motor (corticospinal tracts, midbody of CC) and extra-motor (bilateral anterior cingulate bundles) WM tracts. Moreover, in our population, FA and RD changes in several associative fiber tracts in frontal, temporal and parietal lobes were found to be related to severity of apathy when evaluated by self- and informant ratings. Our results thus suggest that subtle WM changes can be associated with mild behavioral symptoms in ALS even in the absence of overt cognitive and behavioral impairment. Besides allowing the confirmation of previous results, the present study expanded previous analyses towards the use of both self- and informant-rated versions of the AES and of the FrSBe to measure the severity of apathy, enabling the investigation of the potential correlations existing between changes in patients' apathy scores and whole-brain DTI measures.

Microstructural alterations in the CC found in our ALS population were similar to those reported in other cohorts of ALS patients [59, 60]. Similar to previous studies, we performed a TBSS analysis of DTI data, a hypothesis-free technique which is capable of detecting structural changes over the whole brain and therefore highlights the structural process of disease spreading across WM fibers known to connect motor areas [59, 60, 63]. Structural neuroimaging evidence of CC damage seems to provide, *in vivo*, the same neuropathological signatures of neurodegeneration widely reported in both animal models [64] and autopsied patients [65]. Interestingly, executive and behavioral dysfunctions have been shown to be associated mainly with a degeneration of the anterior part of CC (e.g., genu) in several cohorts of ALS patients [63, 65] as well as in FTLD phenotypes, in support of the existence of a frontotemporal lobar degeneration continuum [66]. Moreover, among associative WM tracts in the frontal lobe, the anterior cingulum bundle is adjacent to the motor cortex, and its microstructural damage in ALS has been hypothesized to be related to the pattern of corticofugal disease spreading [67, 68]. The functional involvement of the anterior cingulum in a network of multiple frontal brain regions, which are connected to the basal ganglia and limbic system and which substantially regulate goal-directed and socioemotional behaviors [37, 69–71] and inhibitory control [72], may explain the strong association between the alterations in the anterior cingulum bundle and apathy in both FTLD [69] and ALS [6, 26, 27] patients. Moreover, we observed that not only the FA values in the anterior cingulum bundle, together with FA changes in more widespread WM areas, including several long associative WM tracts, were related to severity of apathy when evaluated by the self-rated apathy subscore of FrSBe. This finding suggests an involvement of more distributed cerebral areas in determining apathy, as also revealed in other neurodegenerative diseases, such as Parkinson's [73] and Alzheimer's [74] diseases, and in neuropsychiatric disorders [75]. Taken together, these results substantially expand the microstructural correlates of apathy and may therefore account for the multidomain structure of this behavioral symptom, which has been previously divided into three different subtypes: cognitive, emotional/affective, and initiation apathy [3]. Indeed, although the apathy subscale of FrSBe allows evaluating apathy as a unitary construct, without excluding the potential confounding effects of motor symptoms on the assessment of this behavioral symptom, a neurobiological model of apathy, known as “the ABC model” [27, 76], currently recognizes three aspects of apathy with different clinical manifestations [23]: (i) affective-emotional apathy, affecting the skill of using emotional context to guide behavior and leading to altered social interactions; (ii) behavioral apathy, which may affect spontaneous patterns of motor movement; and (iii) cognitive apathy, which significantly reduces goal-directed behaviors. Moreover, a valuable overcome of this model has been represented by the “Dimensional Apathy Framework,” which proposed three types of apathy: “initiation, executive, and emotional” apathy [77]. The three types are measured by the DAS, a reliable scale exploring multidimensional apathy in ALS patients by minimising the effects of motor impairment [20, 77]. In this regard, our findings of the relationship between apathy and changes of DTI metrics in more widespread WM areas, beyond the anterior cingulum bundle, deserve to be further investigated in future studies where apathy should be evaluated by DAS. Conversely, the evidence of no significant differences between the subfactors of the AES between the ALS group and healthy subjects and of no correlation between the subfactors of the AES and DTI parameters within the ALS group may further underline the intrinsic limitation of the AES as a one-dimensional apathy measurement scale, with a unitary outcome score, thereby resulting in not being useful for assessing multidimensional apathy [3]. Importantly, Radakovic et al. [78] revealed that in ALS patients, only “initiation” apathy was significantly associated with verbal fluency deficit, while “emotional” apathy was significantly associated with emotional recognition deficits, suggesting that emotional and initiation apathy may have dissociated neural correlates. However, the implementation of the DAS has been limited by its only recent validation in other languages other than English [79]. In particular, at the time of our data collection, the DAS was still not validated in the Italian language, and therefore, we could not employ it in the present study, prospecting to use this scale in future analyses.

When we assessed apathy by the specific subscale of the FrSBe, our findings revealed significant correlations between apathy *T*-scores and FA measures in several associative WM tracts, resembling the findings by Tsujimoto et al. [36], who reported that the severity of apathy was significantly related to FA decrease within the WM underneath the right medial frontal gyrus. Woolley et al. [37] also revealed a significant correlation between changes of apathy subscores of FrSBe and FA decrease in WM underneath bilateral primary motor areas and in the right anterior cingulum bundle and superior longitudinal fasciculus. However, the diverging results obtained by these studies compared to ours might be related to differences in clinical characteristics of the samples included and to methodological differences.

RD measures in the splenium of the CC were found significantly related to the self-rated AES and FrSBe apathy *T*-scores assessed in our ALS population, as also previously described in some non-ALS cohorts of apathetic patients [80, 81]. In particular, Tu et al. [80] reported that the WM damage of the splenium of the CC in Alzheimer's disease and subcortical ischemic vascular disease patients was correlated with the severity of apathy through the involvement of general cognition and dementia severity. Considering that the WM integrity of the body and splenium of the CC is supposed to be related to general cognition on the basis of their numerous fiber projections [82], the reported association between the severity of apathy and the WM integrity of the splenium adds evidence to the notion that widespread WM disconnection and defective cognition may both significantly affect the mechanisms underlying apathy [80]. In support of the abovementioned suggestion, we also found that the informant-rated apathy subscore of FrSBe was inversely related to FA in the WM underneath the left temporal fusiform cortex, which is in line with recent findings concerning the association between apathy scores and frontotemporal regional cerebral blood flow measures in patients with amnestic mild cognitive impairment (aMCI), especially in those with a high probability of being in the aMCI stage of AD [83].

Major limitations of our study are the use of a non-ALS-specific neuropsychological protocol and the recruitment of a small sample size of ALS patients. Specifically, apathy assessment by the subscale of FrSBe, albeit often adopted for ALS patients [4, 36, 57], may be confounded by the presence of motor and bulbar symptoms. Future investigations using ALS-specific neuropsychological protocols, as well as the DAS, to evaluate the multidimensional nature of apathy, should be performed. Moreover, we did not perform a multimodal neuroimaging analysis to combine (and investigate both) WM and grey matter (GM) correlates of apathy. However, when using available T1 MRI data in a standard voxel-based morphometry (VBM) as described previously [60] (data not shown), we did not detect clusters of significant GM atrophy in ALS patients, likely due to the small sample size. Nonetheless, a voxel-wise meta-analysis of 29 VMB studies involving several cohorts of ALS patients revealed that clusters of GM atrophy were mainly located in the right precentral gyrus, the left rolandic operculum, the left lenticular nucleus, and the right anterior cingulate/paracingulate gyri [84], confirming the multisystem nature of ALS, involving extra-motor areas besides the motor system. Of note, multimodal structural approaches, combining both DTI and voxel- or surface-based morphometry measures, have been shown to be more sensitive than each modality separately in the individual ALS patient classification [85, 86], thereby overcoming potential limitations related to each technique for diagnostic purposes.

In conclusion, we identified widespread microstructural correlates of apathy in early stages of ALS. These correlates involve the cingulum bundle and further extend over frontotemporal WM areas. The early involvement of nonmotor areas highlights the multisystem nature of ALS already at the early stages of the disease. Thus, more accurate protocols for the multidimensional assessment of apathy should be implemented routinely in the clinical management of ALS patients. This could help to identify early apathy in ALS, to plan tailored therapeutic interventions, and to improve patients' and caregivers' quality of life.

## Figures and Tables

**Table 1:** Detailed patients and controls characteristics of the patients included in the study.

Parameters	ALS patients mean (SD) (*n* = 21)	Controls mean (SD) (*n* = 19)	*p* (<.05)
*Demographic and clinical measures*			
Age	58.4 (9.83)	57.5 (9.03)	.77
Male/female	13/8	9/10	.35
Education	11.3 (4.6)	11.53 (3.94)	.87
Disease duration (months)	18.71 (11.1)	—	
Disease progression rate	.035 (.037)	—	
ALSFRS-R score	41.3 (3.5)	—	
UMN score	6.1 (4.5)	—	
*Neuropsychological parameters*			
MMSE	28.9 (1.4)	29.72 (1.18)	.06
Digit span test: forward	5.2 (1.5)	5.53 (1.01)	.4
Digit span test: backward	4.2 (1.3)	4.59 (.8)	.4
Token	33.9 (2.08)	35 (1.2)	.07
Memory prose	12.7 (2.96)	14.3 (2.1)	.06
RCPM	27.1 (4.2)	30 (2.82)	.08
Visual discrimination of scrawls	30.4 (1.5)	30.4 (1.3)	.96
SEF	.3 (.7)	.18 (.53)	.52
*Neurobehavioral variables*			
S-AES total score	27.3 (6.7)	25.8 (5.28)	.47
Cognitive factor	12.3 (4.3)	11.5 (2.5)	.51
Behavioral factor	6.9 (1.3)	6.5 (1.5)	.4
Emotional factor	3.4 (1.18)	2.8 (.7)	.09
Other factors	4.6 (1.27)	4.9 (1.4)	.5
I-AES total score	28.6 (7.3)	29.4 (6.3)	.77
Cognitive factor	12.8 (3.6)	13 (2.7)	.9
Behavioral factor	7.2 (1.8)	7.6 (2.7)	.64
Emotional factor	3.5 (1.4)	3.5 (1.5)	.9
Other factors	5 (1.6)	5.2 (1.9)	.78
FrSBe (patient form, total *T*-score)	49.2 (7.8)	—	—
FrSBe (caregiver form, total *T*-score)	57.1 (8.7)	—	—
FrSBe (patient form, apathy *T*-score)	47.9 (9.4)	—	—
FrSBe (caregiver form, apathy *T*-score)	58.1 (10.8)	—	—
BDI-II	11.6 (5.9)	10.18 (8.04)	.57

ALSFRS-R = Amyotrophic Lateral Sclerosis Functional Rating Scale-Revised; S-, I-AES = self-rated, informant-rated Apathy Evaluation Scale; BDI-II = Beck Depression Inventory II; FrSBe = Frontal Systems Behavior; MMSE = Mini-Mental State Examination; RCPM = Raven's Colored Progressive Matrices; SEF = Stroop Executive Factor; UMN = Upper Motor Neuron.

## Data Availability

The MRI and neuropsychological data used to support the findings of this study are available from the corresponding author upon request.
